# Performance of Hybrid Powder-Suspension Axial Plasma Sprayed Al_2_O_3_—YSZ Coatings in Bovine Serum Solution

**DOI:** 10.3390/ma12121922

**Published:** 2019-06-14

**Authors:** Vasanth Gopal, Sneha Goel, Geetha Manivasagam, Shrikant Joshi

**Affiliations:** 1 Department of Physics, School of Advanced Sciences, VIT, Vellore 632014, India; 2 Centre for Biomaterials, Cellular and Molecular Theranostics, VIT, Vellore 632014, India; geethamanivasagam@vit.ac.in; 3 Department of Engineering sciences, University West, 46186 Trollhättan, Sweden; sneha.goel@hv.se (S.G.); shrikant.joshi@hv.se (S.J.)

**Keywords:** axial feeding, hybrid plasma spray coating, bovine serum solution, sliding wear, indentation

## Abstract

Ceramic coatings on metallic implants are a promising alternative to conventional implants due to their ability to offer superior wear resistance. The present work investigates the sliding wear behavior under bovine serum solution and indentation crack growth resistance of four coatings, namely (1) conventional powder-derived alumina coating (Ap), (2) suspension-derived alumina coating (As), (3) composite Al_2_O_3_—20wt % Yittria stabilized Zirconia (YSZ) coating (AsYs) deposited using a mixed suspension, and (4) powder Al_2_O_3_—suspension YSZ hybrid composite coating ApYs developed by axial feeding plasma spraying, respectively. The indentation crack growth resistance of the hybrid coating was superior due to the inclusion of distributed fine YSZ particles along with coarser alumina splats. Enhanced wear resistance was observed for the powder derived Ap and the hybrid ApYs coatings, whereas the suspension sprayed As and AsYs coatings significantly deteriorated due to extensive pitting.

## 1. Introduction

Thermal spray technique is one of the most versatile surface engineering processes and enables the production of protective coatings, especially for harsh environments such as wear, corrosion/oxidation, high temperature, etc. Among the different variants within the thermal spray family, plasma spraying is a well-established technique used in many applications, such as aerospace, automotive, marine, and medical devices [1,2]. Particularly, plasma spraying has an important role in orthopaedic implant application, where it is used to develop bio-ceramic (hydroxyapatite) coatings on the hip femoral stem to enhance Osseo-integration. Furthermore, the hydroxyapatite coating on the hip femoral stem is approved by the Food and Drug Administration (FDA) in the USA [3,4]. Plasma spraying involves the use of high energy plasma to transform the powder particles of the coating material into their molten state, and these are then propelled towards the substrate to form a coating. Usually, conventional plasma spraying uses a powder feedstock with a particle size distribution in the range of 10–100 μm, which results in a relatively coarse structured coating. Recently, the deposition of finely structured coatings using plasma spraying has gained attention due to their ability to yield improved performance [5,6,7]. However, the production of such finely structured coatings using plasma spraying has traditionally been a huge challenge due to the inherent flowability problems associated with the use of fine feedstock powders [8].

Suspension plasma spraying (SPS) has now emerged as an attractive technique to produce fine structured coatings. The process involves suspending a fine sub-micron or nanometric powder in a solvent and injecting the resulting suspension into a plasma flame to overcome the above issue of flowability associated with fine powders [9,10]. Axial suspension feeding is a recent advancement in the SPS process and involves the suspension being injected axially into the plasma flame rather than radially. This axial feeding enhances the enthalpy exchange between the plasma flame and the sprayed material [11] and allows more efficient utilization of the plasma energy that is essential to achieve industrially relevant throughputs. The advent of the SPS process with axial feeding provides an opportunity to combine coarse powder feedstock and a fine structured suspension feedstock to realize hybrid coatings with superior properties. In the present study, four different coatings were developed using axial suspension plasma spraying. These included a conventional monolithic spray grade powder derived alumina coating (Ap) and a pure suspension-derived alumina coating (As). These were compared with two composite alumina—yttria-stabilized zirconia (YSZ) coatings, one derived from a mixed suspension (AsYs) and the other deposited using a hybrid feedstock (ApYs), in which a conventional micron-sized alumina powder and fine sized YSZ in the form of suspension were axially injected simultaneously into the plasma flame. The coating developed by the hybrid process possesses a multi-scale structure, i.e., the presence of both micron- and fine-sized features, due to typical splat sizes that result from the conventional coarse spray-grade powder and the much finer powder that constitutes the suspension [12]. The choice of alumina and YSZ as an illustrative material system to demonstrate the efficacy of hybrid coatings was based on the combination of high hardness and Young’s modulus of the Al_2_O_3_ matrix with an additional toughening effect expected from the YSZ dispersion, thereby significantly increasing the flexural strength and the fracture toughness of Al_2_O_3_ [13,14,15]. Furthermore, the application of zirconia toughened alumina in hip implants has been already reported to significantly reduce the failure rate of implants due to the superior wear resistance [16,17].

Although the zirconia toughened alumina hip implants have significantly reduced the failure rate, the sudden fracture of bulk ceramic implants has raised inevitable concerns over their utility. Consequently, ceramic coatings on metallic implants are a promising alternative due to their ability to offer superior wear resistance, with the metallic substrate providing good fracture toughness. Considering the impact of the wear and the fracture toughness on the longevity and the performance of hip implants, the present study aimed to investigate the sliding wear behaviour of all the above-mentioned ceramic coatings in the presence of a bovine serum albumin solution. In addition to wear resistance, the indentation crack growth resistance of the developed coatings was also investigated, and the prominent results are presented herein.

## 2. Experimental Methods

### 2.1. Substrate Materials, Powders, and Suspensions

The suspensions used in the study were of Al_2_O_3_ (d10 = 0.51 µm, d50 = 2.20 µm, d90 = 4.93 µm) for As and 8 wt % YSZ (d10 = 230 nm, d50 = 440 nm, d90 = 950 nm) for ApYs coating. A mixed suspension of the above two to obtain an Al_2_O_3_-20wt %YSZ composition was used in the case of the AsYs coating. All the suspensions were procured from the same supplier (Treibacher, Austria) with identical solid load content of 25 wt % in ethanol. Spray-grade powders of Al_2_O_3_ (AMPERIT^®^ 740, fused and crushed, 22/5 µm, H.C. Starck GmbH, München, Germany) and NiCoCrAlY (AMPERIT^®^ 410, gas atomized, H.C. Starck GmbH, München, Germany) were also used as feedstock materials. The substrate material employed was Domex 355 steel for all the coatings. Although this material is not specifically intended for biomedical applications, it was used out of convenience since, in the case of overlay coatings such as those that are plasma sprayed, the investigated wear resistance properties of the deposited layer are not expected to be influenced by the substrate.

### 2.2. Coating Deposition

**Figure 1:**
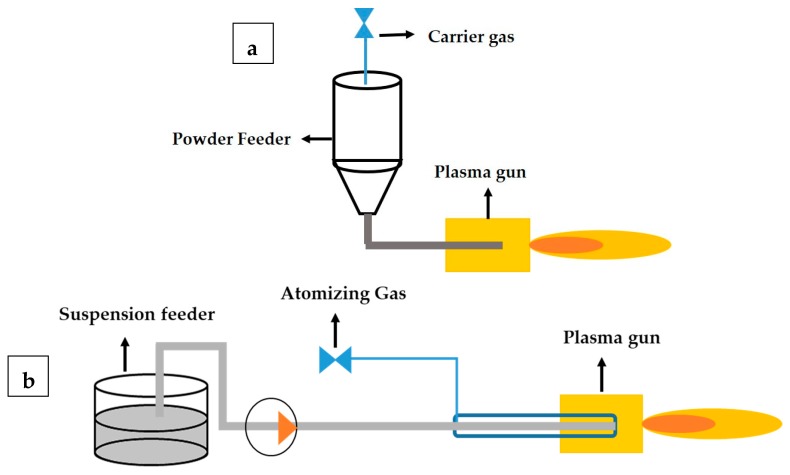

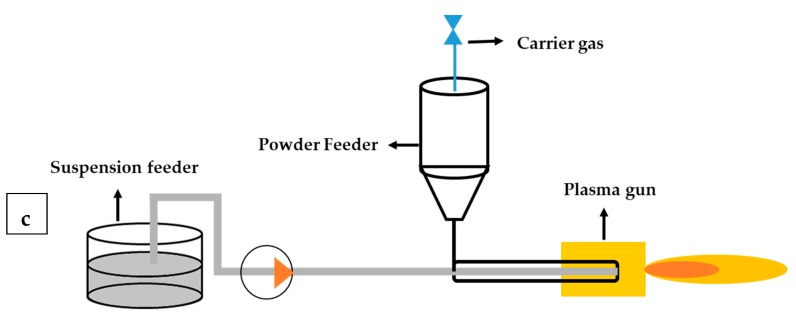
Schematic representation of the axial plasma spraying process of (**a**) Ap, (**b**) As and AsYs (**c**) ApYs coatings.

All the coatings produced in this study were sprayed using a Mettech Axial III high power plasma torch (Northwest Mettech Corp., Vancouver, BC, Canada) capable of the axial introduction of feedstock and equipped with a Nanofeed 350 suspension feeder as well as a separate powder feed system (Uniquecoat, model PF50WL, Richmond, VA, USA). The plasma spray process involved in the current study has three different processes:

(a) The Axial feed of conventional powder to develop Ap coating. In this process, the feedstock powder was carried inside a tube and injected just before the plasma gun exit. Argon was used as a carrier gas to transform the powder from the powder feeder to the plasma torch.

(b) The second process involved the axial feed of suspension with a coaxial feed of atomizing gas to develop As and AsYs coatings. Instead of the simple tube in the case of Ap coating, a larger tube housing a coaxially mounted liquid feedstock injector was used in suspension spraying. The inner tube carried the suspension while surrounding the inner tube; an atomizing gas was fed into the annular region to atomize the feedstock. The liquid feedstock was pumped into the plasma torch, and the feed rate could be varied by varying the atomizing gas.

(c) The third process involved the combination of the above two processes to fetch the hybrid powder-suspension ApYs coating. The process involved the axial feed of suspension with a coaxial feed of powder with a carrier gas. The amount of suspension, powder feed rate, and carrier/atomizing gas could be controlled individually. The process parameters employed for depositing the coatings As, AsYs, Ap, and ApYs are given in Table 1. A schematic representation of the plasma spray process involved in the current study is depicted in Figure 1. Prior to the coating, the substrates were grit blasted with alumina grit of 80 μm size to induce surface roughness, which enhanced the mechanical interlocking between coating and substrate. Furthermore, the grit blasted substrates were the first bond coated with NiCoCrAlY to enhance the adhesion of the coatings.

### 2.3. Coating Characterization

Microstructural investigation of polished cross-sections of the coatings was carried out using SEM (HITACHI, TM3000, Tokyo, Japan). The phase constitution of the composite coatings was analyzed using a Siemens D500 XRD with Cr Kα radiation (λ = 2.29 Å) with the diffraction angle (2θ) varied between 10° and 120°. In addition, an energy dispersive spectroscopy (EDS) system (EDAX, Mahwah, NJ, USA) was utilized for composition analysis of cross-sections of the coating. The porosity of the coatings was measured using an image analysis technique ImageJ software (National Institute of Health, USA). Twenty cross-sectional SEM images were taken at appropriate magnification (×500) for porosity measurement. The grey scale images were converted into black and white by manual threshold adjustment. The average porosity values of the coatings were given in the table.

### 2.4. Mechanical Characterization

#### 2.4.1. Vickers Micro Hardness

The hardness of all the coatings was measured using Vickers micro-hardness testing (HMV-2, Shimadzu Corp., Kyoto, Japan) with a constant load of 100 g and a dwell time of 15 s. A total of eight indents were recorded on each specimen, and the average hardness and the standard deviation were determined.

#### 2.4.2. Indentation Crack Growth Resistance

**Figure 2:**
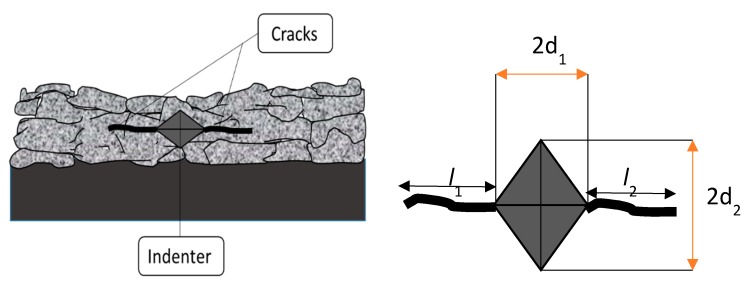
Schematic representation of crack propagation from Vickers indentation and the measurement of crack length.

The crack growth resistance of the coatings was evaluated by Vickers indentation method. The indentations were made on polished coating cross sections. The indenter was carefully positioned in such a way that the indent was made approximately at the centre of the coating. A load of 2 kg was chosen in the case of all the coatings, since lower loads were not found to lead to any discernible crack formation. A minimum of five indentations was carried out, and the lengths of the cracks were measured using an optical microscope (Carl Zeiss). The crack length *C* was measured using the equation [18]:

$$C=\frac{\left(2d_{1}+2d_{2}\right)}{4}+\frac{\left(l_{1}+l_{2}\right)}{2}$$

where 2*d*_1_ and 2*d*_2_ are the parallel and the perpendicular Vickers indenter diagonals, *l*_1_ and *l*_2_ are the lengths of the cracks observed on either side of the indent parallel to the lamellae, depicted to originate below in Figure 2 from the left and the right side of the indent corners. A schematic representation of crack propagation from the Vickers indenter corner and the measurement of the crack length are shown in Figure 2 below:

### 2.5. Sliding Wear Test

The coatings were subjected to a sliding wear test carried out using a linearly reciprocating wear tester TR-285 M machine (DUCOM, Bengaluru, India) as per American Society for Testing and Materials (ASTM) standard G133-05 [19]. An alumina ball with a diameter of 6 mm was used as the counterpart. The wear tests were carried out in the presence of a 4 mg/mL bovine serum albumin solution with the addition of 0.2% sodium azide to retard bacterial growth. The whole test set up was maintained at 37 °C for 10^5^ cycles. Prior to the wear test, all the coatings were polished using SiC emery sheets (600 to 3000 grit size) and finally polished with 0.1 μm diamond paste. The polished coatings were ultrasonically cleaned in acetone to get rid of any contaminants. The wear volume was calculated by following the ASTM G133-05(2016) standard [19] using the relationship *V* = *A* × *l*, where *A* is the average cross-sectional area of the track (mm^2^) and *l* length of the stroke (mm). The area of the wear track was measured using a three-dimensional (3-D) optical profilometer (Taylor Hobson, Leicester, UK). The specific wear rate was measured using the following formula:

$$WR=\frac{V}{L\times S}$$

where *WR* represents wear rate (mm^3^ N^−1^ m^−1^), *V* is the wear volume (mm^3^) determined as the product of the mean worn area and the width of wear track, *L* corresponds to normal load (N), and *S* signifies sliding distance (m). 

## 3. Results and Discussion

### 3.1. Cross-Sectional Morphology

**Figure 3:**
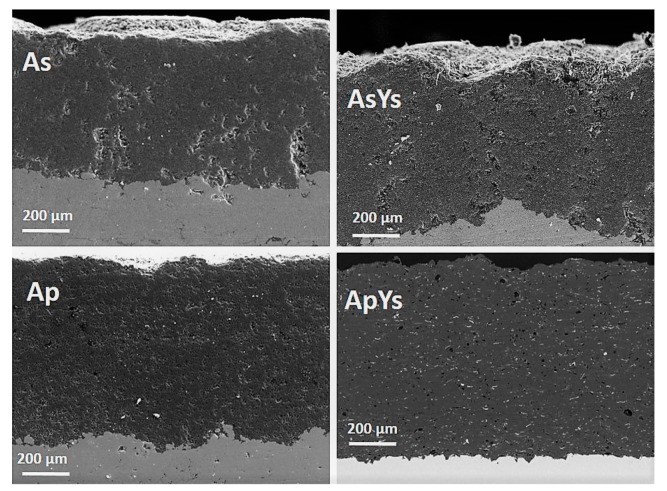
Cross-sectional SEM images of As, AsYs, Ap, and ApYs coatings.

Figure 3 shows the cross-sectional morphology of the four coatings, As, AsYs, Ap, and ApYs. All the coatings revealed a well-intact interface with the bond coat, which was indicative of good adhesion.

**Figure 4:**
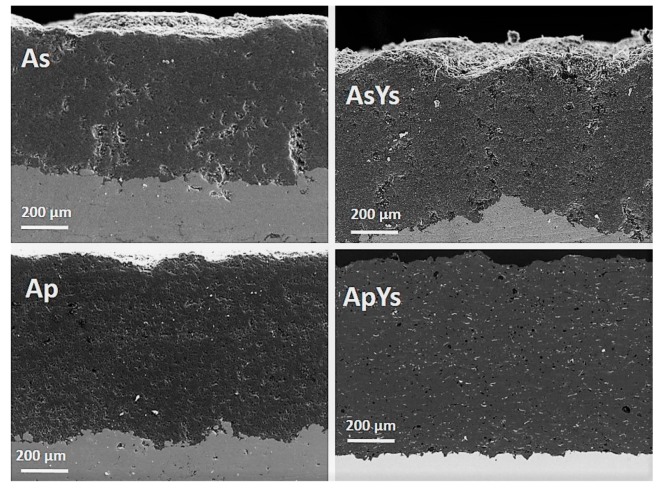
Cross-sectional SEM images of As, AsYs, Ap, and ApYs coatings taken at higher magnification.

**Figure 5:**
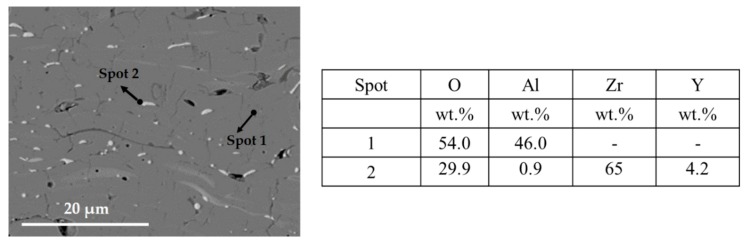
Energy dispersive spectroscopy (EDS) analysis on the cross-sectional SEM image of hybrid ApYs coating and its elemental composition taken at two different spots confirms the bright region to be Zr.

Figure 4 shows higher magnification images of the coatings AsYs and ApYs, which revealed the incorporation of YSZ (seen as the brighter phase) in the coating over a large area. Particularly, the incorporation of YSZ was finer and more homogenous in the AsYs coating compared to the ApYs coating, plausibly due to the significantly smaller particle size of the Alumina splats comprising the matrix. In addition, EDS analysis was carried out on the cross-section of the ApYs coating to confirm the presence of the Zr region. Spot 1, which was taken at the darker area, confirmed the presence of Al_2_O_3_, whereas spot 2 at the brighter region confirmed the presence of a Zr rich region, as shown in Figure 5. The percentage porosities of all the coatings were determined by image analysis (using ImageJ software) and are given in Table 2. The porosity of the AsYs (3.5 ± 2.2 vol%) was found to be the highest among all the coatings, whereas the porosities of Ap, As, and ApYs coatings showed no significant difference.

### 3.2. XRD Analysis

**Figure 6:**
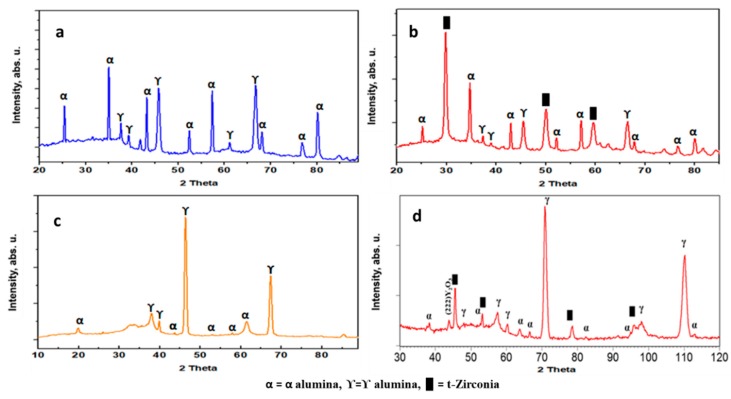
XRD spectra of coatings (**a**) As, (**b**) AsYs, (**c**) Ap, and (**d**) ApYs.

The XRD patterns of the coatings (As, AsYs, Ap, and ApYs) are shown in Figure 6. The conventional alumina coating Ap exhibited the typical XRD pattern representative of conventional plasma sprayed alumina coatings in which the metastable γ-Al_2_O_3_ was observed to be the major phase along with traces of α-Al_2_O_3_ phase_._ It is well known that in conventional plasma spraying, the presence of γ-Al_2_O_3_ is attributable to the rapid splat quenching of alumina during the plasma spray process, whereas the α-Al_2_O_3_ results from partially melted or unmelted alumina particles [20,21,22,23,24]. On the contrary, the coating As which was produced by the SPS technique showed α-Al_2_O_3_ as the predominant phase rather than γ-Al_2_O_3_. The disparity in the alumina phase constitution between the conventional atmospheric plasma spraying and suspension spraying is that, in the conventional spraying, the alumina particles are in direct contact with the plasma flame, which subjects the particles to extensive melting and subsequent rapid splat quenching, leading to the formation of a significant amount of γ-Al_2_O_3_ phase. In the latter case, the evaporation of the solvent consumes a substantial portion of the energy available in the plasma flame, which plausibly results in lower particle temperature at impact, leading to reduced cooling rates that promote the retention of the α-Al_2_O_3_ phase. In this context, it is also relevant to mention that a prior study already demonstrated the formation of phase-pure α-Al_2_O_3_ using a liquid feedstock involving a solution precursor rather than a suspension [25]. In the case of both the all-suspension composite AsYs coating and the powder-suspension hybrid composite ApYs coating, the presence of tetragonal ZrO_2_ distributed in Al_2_O_3_ was confirmed. Furthermore, all the coatings that involved the use of a suspension feedstock showed a broad hump in 2theta position from approximately 20° to 60°, which was suggestive of the presence of amorphous phases in the coatings.

### 3.3. Hardness

**Figure 7:**
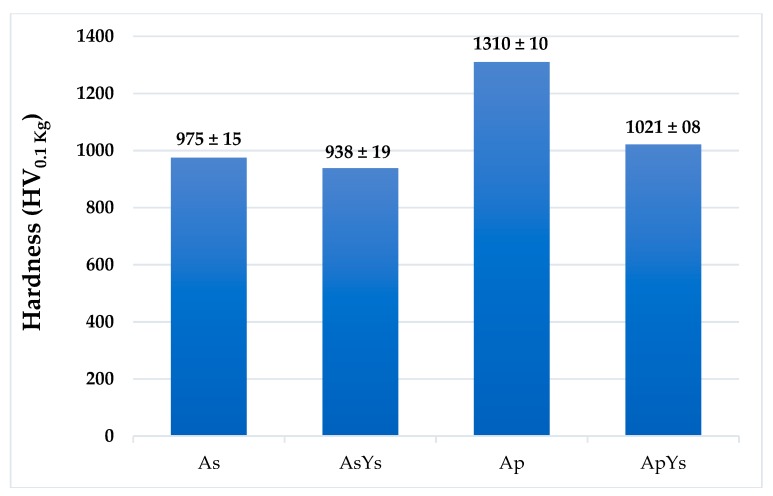
Vickers microhardness values of the coatings As, AsYs, Ap, and ApYs.

Figure 7 shows the Vickers microhardness of As, AsYs, Ap, and ApYs coatings, respectively. Among all the powder-derived coatings, Ap exhibited the highest hardness of 1310 ± 15 HV followed by the hybrid coating ApYs 1021 ± 19 HV. Another interesting result was that the suspension coating As exhibited lower hardness compared to the conventional powder Ap coating. This lower hardness could be attributable to the inherent differences in the microstructures of the powder-derived and the suspension-derived coatings in terms of individual splat sizes, a number of splat boundaries, porosity and pore distribution, etc. It was also noted that the suspension derived coating AsYs exhibited the lowest hardness 938 ± 08 HV among all the coatings. A relative decrease in hardness in the cases of ApYs and AsYs compared to Ap and As respectively was due to the inclusion of YSZ, which has an intrinsic lower hardness than alumina [22].

### 3.4. Indentation Crack Growth Resistance

**Figure 8:**
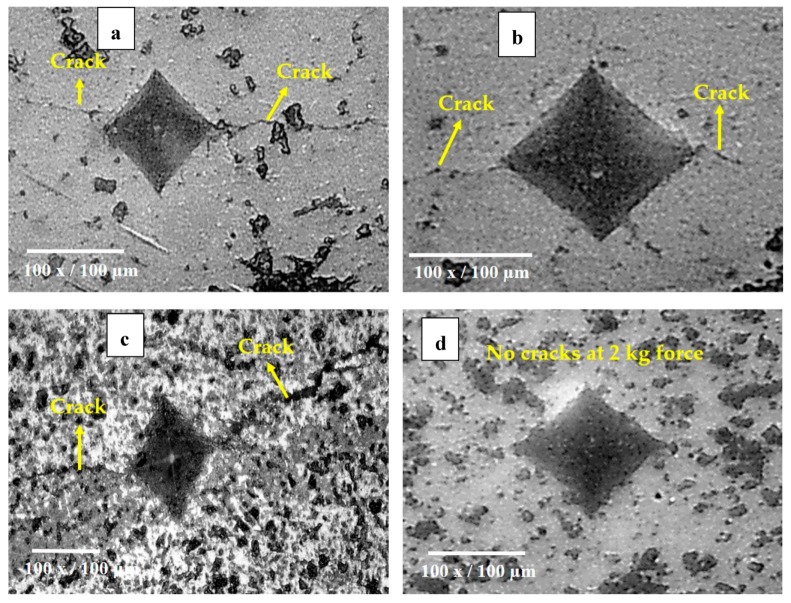
Optical micrographs revealing indentation crack growth in (**a**) As (**b**) AsYs (**c**) Ap and (**d**) ApYs.

The Vickers indentation technique is a versatile tool for assessing the fracture toughness of bulk brittle materials—particularly ceramics. However, the determination of fracture toughness of plasma sprayed coatings employing this technique is quite complicated due to the highly anisotropic microstructure associated with the typical lamellar structure [26]. Therefore, in the present work, the indentation technique was utilized to study the crack growth resistance of the coatings rather than for fracture toughness measurement. A similar approach of investigating the indentation crack growth resistance by measuring the crack length was reported previously by Hong Luo et al. [26]. Figure 8 shows the indentation crack growth in As, AsYs, Ap, and ApYs coatings. The crack length was measured and is tabulated in Table 3. From Figure 8, it is evident that the coatings As and Ap showed a long crack originating from the two indent corners parallel to the coating–substrate interface. However, there were no cracks observed from the indentation perpendicular to the coating–substrate interface. This asymmetric crack behaviour was due to the inherent splat microstructure of the plasma sprayed coatings [26]. On the contrary, the hybrid coating ApYs did not show any cracks around the indentation either parallel or perpendicular to the coating–substrate interface, whereas the coating AsYs exhibited short cracks along the plane of the coating–substrate interface.

The crack growth resistance of a coating was evaluated from the length of the cracks that emerged from the two indenter corners parallel to the coating–substrate interface. The smaller the crack length, the larger the crack growth resistance. In this context, the hybrid coating ApYs was observed to possess superior crack growth resistance with no crack originating from the indenter corners, even at 2 kg force. The enhanced crack growth resistance of the ApYs coating could plausibly be attributed to one or more of the following: (i) the intrinsic phase transformation toughening associated with tetragonal-to-monoclinic transition of zirconia phase, which restricted crack propagation [16], or (ii) the inclusion of finer YSZ particles in coarser alumina splats leading to an enhancement in cohesion strength between the splats and thereby resulting in superior crack growth resistance. In the case of AsYs coatings, considerably smaller cracks were observed to have emanated at the indenter corners compared to As, which highlights the role of the finer YSZ included in the former coating in enhancing the crack growth resistance. According to the measured crack length, the indentation crack growth resistance of the coatings could be ranked as ApYs > AsYs > As > Ap. The inferior crack growth resistance of the powder derived Ap was due to the presence of γ-Al_2_O_3_ phase. It has been reported that the γ-Al_2_O_3_ phase present in the high velocity oxy fuel (HVOF) sprayed alumina coating worsened the fracture toughness compared to the mixed α and γ-Al_2_O_3_ phases [27].

### 3.5. Sliding Wear

The sliding wear behaviours of As, AsYs, Ap, and ApYs coatings were investigated in the presence of bovine serum albumin solution. In order to understand the prevailing wear mechanisms, the coatings were grouped into different categories. The coatings Ap–As were grouped to study the wear performance of conventional powder-derived and fine structured suspension-derived coatings. Coatings Ap–ApYs were compared to evaluate the effects of the introduction of a fine structured second phase on the performance of a conventional coarse-structured Ap coating. Similarly, coatings As–AsYs were also compared to investigate how the introduction of fine structured YSZ second phase affected the performance of the fine structured As coating.

#### 3.5.1. Specific Wear Rate

**Figure 9:**
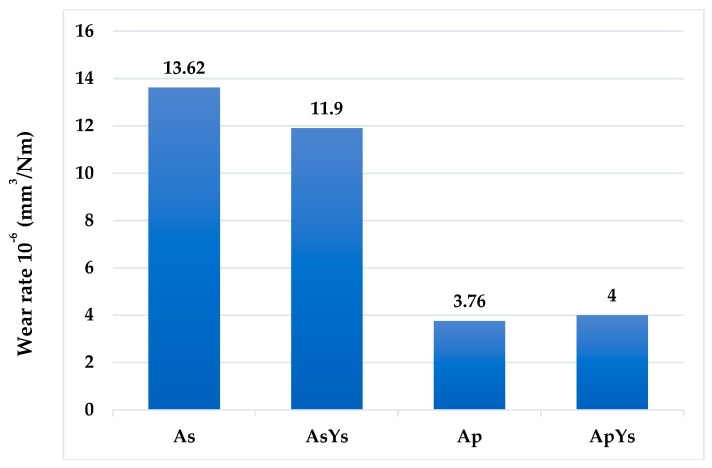
Specific wear rates of As, AsYs, Ap, and ApYs coatings.

**Figure 10:**
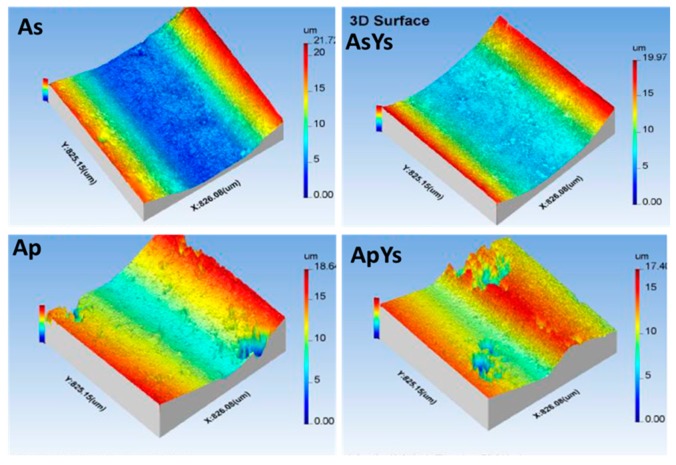
Three-dimensional (3-D)-optical profilometer images of coatings As, AsYs, Ap, and ApYs.

The specific wear rates of the coatings were calculated and are shown in Figure 9. The wear rate of the Ap coating was found to be the lowest among all the coatings. In contrast, the wear rate of the As coating was highest among all the coatings. In the case of the hybrid ApYs coating, the wear rate was almost similar to that of the Ap coatings. The wear rate of AsYs was slightly lower than As but significantly higher compared to Ap and ApYs coatings. Further, the wear depth assessed by 3-D optical profilometer in Figure 10, showed larger wear depth for coatings As and AsYs, whereas the wear depth was smaller for Ap and ApYs coatings. According to the wear rate values determined, the wear rate of the investigated coatings could be ranked in the order Ap ≈ ApYs < AsYs < As.

#### 3.5.2. Worn Surface Morphology

**Figure 11:**
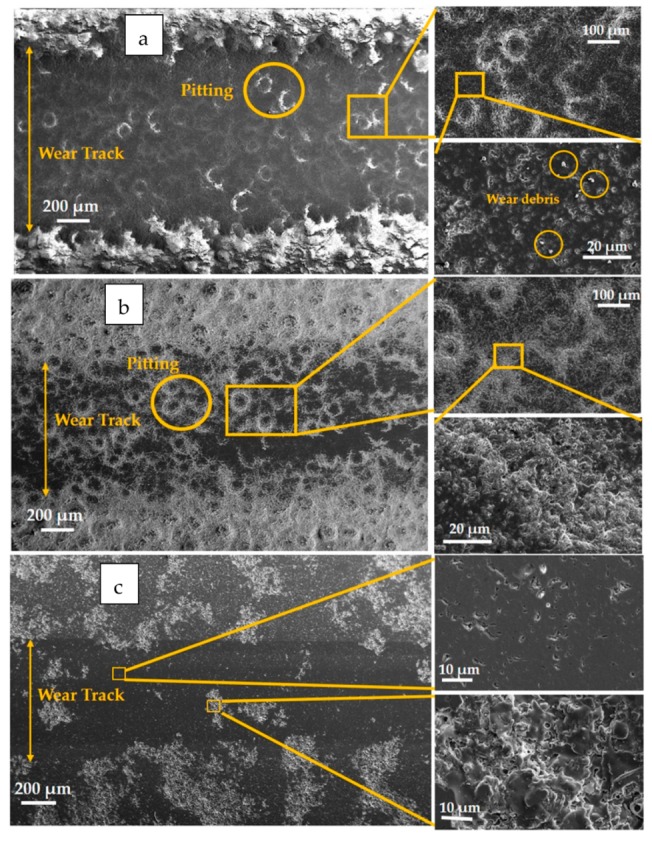

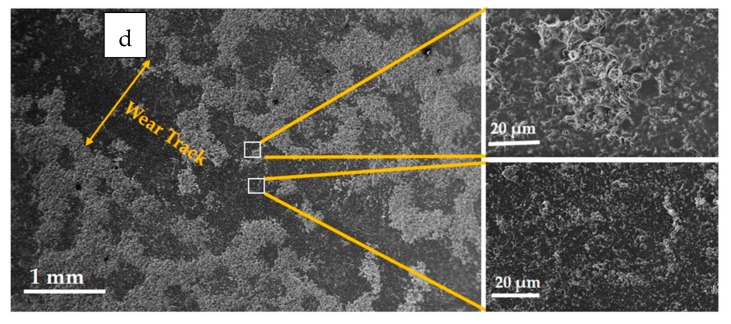
Worn surface morphologies at different magnifications in the case of (**a**) As, (**b**) AsYs, (**c**) Ap, and (**d**) ApYs coatings.

Figure 11 shows the worn surface morphology of all the coatings. The worn surface of the Ap coating revealed a smooth surface. Higher magnification images showed uniformly distributed pits with no evidence of fine grooves or wear debris. Furthermore, in the wear track, underlying splats unaffected during sliding were also observed. In comparison, the worn surface morphology of the As coating revealed substantial pitting formation with a rougher surface. Furthermore, at higher magnification, the presence of nano-sized wear debris was also observed. When comparing ApYs and AsYs coatings, the latter showed extensive pitting with a rough, worn surface, whereas the ApYs coatings showed occasional pitting. From the above results, it could be inferred that the suspension derived coatings As and AsYs underwent severe pitting in serum solution, whereas the powder derived Ap and hybrid ApYs coatings exhibited occasional pitting. The extent of pitting directly influenced the wear resistance of the coatings. The coatings As and AsYs, which showed extensive pitting, possessed a higher wear rate; particularly, the As coating possessed the highest wear rate among all the coatings. In contrast, the wear rates of coatings Ap and ApYs were significantly reduced.

As suggested by Hawthrone et al. [28], the wear of thermal spray coatings occurs via three processes, (i) microchipping and ploughing, (ii) debonding at splat boundaries, and (iii) splat fractures associated with porosity. In the case of coatings with poor inter-splat bonding, the primary material removal mechanism is debonding at splat boundaries and results in an increased wear rate. 

**Figure 12:**
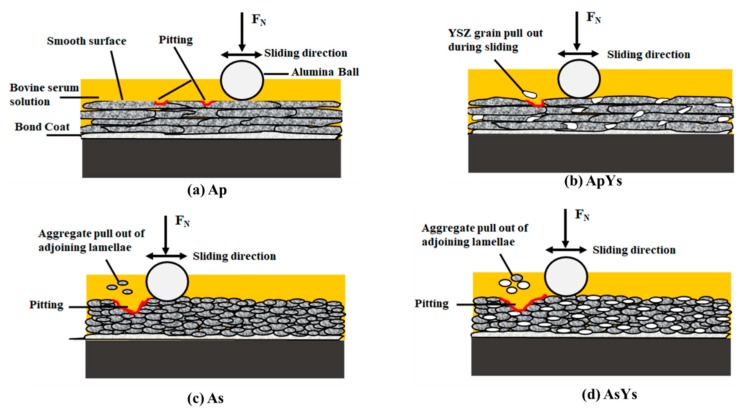
Schematic representation of wear of all the coatings in bovine serum solution.

Additionally, many prior reports have shown that the wear resistance of plasma sprayed ceramic coatings increases with enhanced hardness and toughness [29,30,31]. In this context, the Ap coating was found to possess the highest hardness among all the coatings, which explains its superior wear resistance. It is noteworthy to mention that a previous study involving one of the authors showed that the coating Ap under dry sliding conditions possessed the highest specific wear rate among all the four coatings [32]. However, in the present study, the wear rate of the Ap coating was the least among all the coatings. This reduction in wear rate could be easily explained by the introduction of lubricant. Also, the presence of proteins in bovine serum solution could significantly influence the frictional and the wear behaviours. Many authors have reported that, under bovine serum lubrication, a tribolayer could be formed on the surface, which limits direct contact between mating materials, thereby reducing friction and wear [33,34]. Furthermore, the underlying splats that are not affected during sliding may act as reservoirs for the lubricant. During sliding, the lubricant tends to be drawn up to spread on the surface, leading to a reduction in friction, wear, and seizure [35]. This could also explain the absence of grooves and grain pull outs in the case of Ap. A schematic wear mechanism of all the coatings in the presence of bovine serum solution is shown in Figure 12.

In the case of the hybrid ApYs coating, even though the hardness was lower compared to that of the Ap coating, the indentation crack growth resistance was enhanced by the inclusion of much finer YSZ, with the splat sizes of alumina and YSZ in ApYs differing by nearly two orders of magnitude. This combination of optimum hardness and enhanced fracture toughness could significantly reduce the brittle fracture, thereby resulting in a wear rate that was comparable to that of the Ap coating. In contrast, the suspension derived coatings (As and AsYs) exhibited relatively poor wear resistance. Furthermore, the worn surface morphology of the As and the AsYs coatings suggested that pitting in the presence of the bovine serum solution was a predominant damage mechanism and could have resulted in severe wear. It is essential to mention that the powder derived Ap and hybrid ApYs coatings were also found to have undergone pitting, but the extent was much lower compared to the As and the AsYs coatings. The formation of pitting of ceramic prosthesis under bovine serum solution was previously reported by Rainforth et al. [36]. They reported that the Biolox Delta (Zirconia toughened alumina ceramic hip prosthesis) underwent severe pitting in serum solution when compared to ultra-pure water and Carboxy Methyl Cellulose (CMC)-Na solution as a lubricant. The initiation of pitting was primarily due to the intergranular fracture and the grain pull-out with the formation of a localized region of craters. Furthermore, they also observed that the pits were first initiated from the removal of zirconia grains followed by some of the alumina grains. Similarly, the coatings As and AsYs, which had lower hardness and toughness compared to Ap and ApYs coatings, might have been subjected to intergranular fracture and grain pull-out during sliding, which substantiated the formation of pitting. Moreover, the extensive pitting of AsYs compared to the As coating could have been attributed to the removal of zirconia grains followed by alumina grains. Furthermore, it was supposed that the difference in the splat size of As and AsYs, which was approximately ten orders lesser than the Ap and the ApYs coatings, attributed to the extensive pitting. During sliding, aggregate pull out of adjoining lamellae took place and led to extensive pitting of the As and the AsYs coatings. On the other hand, the Ap and the ApYs coatings possess superior wear resistance behaviours under bovine serum solution despite showing local pitting formation. 

### 3.6. The Coefficient of Friction

**Figure 13:**
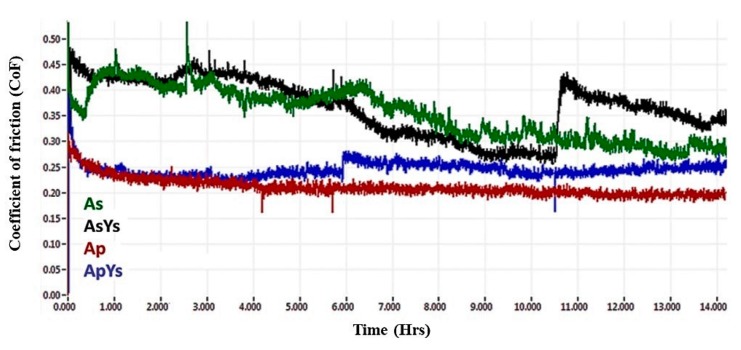
Coefficients of friction of As, AsYs, Ap, and ApYs coatings determined during wear testing in the presence of bovine serum.

The evolution of the coefficients of friction (CoF) of Ap, As, AsYs, and ApYs coatings during wear tests conducted in the presence of bovine serum are shown in Figure 13. As expected, the Ap and the hybrid ApYs coatings exhibited low CoF values compared to the As and the AsYs coatings. The average CoF values of the Ap and the ApYs coatings were 0.21 and 0.24, respectively. In the cases of As and AsYs, the CoFs were found to be higher at 0.34 and 0.36, respectively.

The reason for lower CoF of the Ap coating was attributed to the microstructure of the coating. The conventional powder alumina coating yielded typically smooth, disk-like splats (fully molten splats), which usually resulted in lower local surface roughness. In addition, the polished, worn surface (see Figure 11c) and the presence of proteins in bovine serum solution also attributed to the lower CoF values of the Ap coating. Similarly, the hybrid coating ApYs also showed only marginally higher CoF values compared to the Ap coating despite showing a slightly rougher worn surface. In the case of the pure suspension coating As and the mixed suspension coating AsYs, the CoFs were found to be higher. As already seen in Section 3.5, the worn surface morphology of the pure suspension coatings (As and AsYs) revealed a rougher surface due to extensive pitting, which could explain the higher CoF values. 

## 4. Conclusions

Alumina-YSZ ceramic composite coatings deposited using a hybrid powder-suspension feedstock (ApYs) were studied, and their microstructures and mechanical properties were compared with conventional powder-derived (Ap) and suspension-derived (As) alumina coatings, as well as coatings deposited using mixed alumina–YSZ suspension (AsYs). The sliding wear and the indentation crack growth resistance of these coatings were explicitly investigated, and the critical conclusions that could be drawn are as follows:

- The hardness of the conventional monolithic alumina coating Ap was the highest among all the coatings, which was 1.28 times higher than the hybrid ApYs coating, 1.34 times higher than the As coating, and 1.39 times higher than the AsYs coating. The inclusion of YSZ in both Ap and As coatings lowered the hardness by virtue of its lower intrinsic hardness.
- The indentation crack growth resistance of the hybrid coating ApYs was superior when compared to all the other coatings. The inclusion of finer YSZ particles into the coarser alumina splat increased the cohesion strength and resulted in superior crack growth resistance.
- The worn surface morphology of the Ap coatings exhibited polishing wear without any grooves, whereas the hybrid coating ApYs coating revealed local pitting. In the case of the pure and the mixed suspension coatings (As and AsYs), extensive pitting was observed, which deteriorated the wear resistance properties.

Overall, the hybrid coating ApYs showed promising results in terms of superior indentation crack growth resistance without any compromise in wear resistance properties compared to the other coatings. The optimum wear resistance and the superior indentation crack growth resistance of the hybrid ApYs coating could be a potential candidate for implant application. On the other hand, the suspension derived coatings (As and AsYs) showed detrimental properties by exhibiting severe pitting during sliding under the bovine serum solution. 

## Figures and Tables

**Table 1:** Process parameters employed for depositing As, AsYs, Ap, and ApYs coatings.

Parameters	ApYs	Ap	As	AsYs
Operating gas	H_2_, N_2_	H_2_, N_2_	H_2_, N_2_, Ar	H_2_, N_2_, Ar
Spray Distance, mm	150	150	100	100
Current, A	230	230	220	220
Powder feed rate, g/min	40	50	-	-
Suspension feed rate, mL/min	40	-	40	40
Power, kW	124	109	122	122

**Table 2:** Percentage porosity values of the coatings.

Coatings	As	AsYs	Ap	ApYs
Porosity (Vol%)	2.5 ± 0.9	3.5 ± 1.2	2.2 ± 0.3	2.6 ± 0.3

**Table 3:** Indentation crack length in the coatings.

Coatings	Crack Length (µm)
As	111.35
AsYs	54.72
Ap	181.50
ApYs	No visible cracks

## References

[1] Saber-Samandari S., Berndt C.C. (2013). IFTHSE Global 21: Heat treatment and surface engineering in the twenty-first century Part 10–Thermal spray coatings: A technology review. Int. Heat Treat. Surf. Eng..

[2] Plasma Spray Coating Thermal Spray Coating Thermal Spray Technologies Inc.. http://www.tstcoatings.com/plasma_spray.html.

[3] Ivanka I., Vladislav A., Christoph M.S., Hristo K.S., Boyko G. (2012). Plasma Sprayed Bioceramic Coatings on Ti-Based Substrates: Methods for Investigation of Their Crystallographic Structures and Mechanical Properties. Advanced Plasma Spray Applications.

[4] Chu P.K., Chen J.Y., Wang L.P., Huang N. (2002). Plasma-surface modification of biomaterials. Mater. Sci. Eng. R..

[5] Zhou C., Wang N., Wang Z., Gong S., Xu H. (2004). Thermal cycling life and thermal diffusivity of a plasma-sprayed nanostructured thermal barrier coating. Scr. Mater..

[6] Chen H., Zhou X., Ding C. (2003). Investigation of the thermomechanical properties of a plasma-sprayed nanostructured zirconia coating. J. Eur. Ceram. Soc..

[7] Liang B., Ding C. (2005). Thermal shock resistances of nanostructured and conventional zirconia coatings deposited by atmospheric plasma spraying. Surf. Coat. Technol..

[8] Brinley E., Babu K.S., Seal S. (2007). The solution precursor plasma spray processing of nanomaterials. JOM.

[9] Ganvir A. (2014). Comparative Analysis of Thermal Barrier Coatings Produced Using Suspension and Solution Precursor Feedstock. Master’s Thesis.

[10] Fauchais P., Montavon G., Lima R.S., Marple B.R. (2011). Engineering a new class of thermal spray nano-based microstructures from agglomerated nanostructured particles, suspensions and solutions: An invited review. J. Phys. Appl. Phys..

[11] He P., Sun H., Gui Y., Lapostolle F., Liao H., Coddet C. (2015). Microstructure and properties of nanostructured YSZ coating prepared by suspension plasma spraying at low pressure. Surf. Coat. Technol..

[12] Björklund S., Goel S., Joshi S. (2018). Function-dependent coating architectures by hybrid powder-suspension plasma spraying: Injector design, processing and concept validation. Mater. Des..

[13] Chevalier J., Aza A.H.D., Fantozzi G., Schehl M., Torrecillas R. (2000). Extending the Lifetime of Ceramic Orthopaedic Implants. Adv. Mater..

[14] Kern F., Palmero P. (2013). Microstructure and mechanical properties of alumina 5 vol% zirconia nanocomposites prepared by powder coating and powder mixing routes. Ceram. Int..

[15] Mangalaraja R.V., Chandrasekhar B.K., Manohar P. (2003). Effect of ceria on the physical, mechanical and thermal properties of yttria stabilized zirconia toughened alumina. Mater. Sci. Eng. A.

[16] Kurtz S., Kocago S., Arnholt C., Huet R., Ueno M., Walter W.L. (2014). Advances in zirconia toughened alumina biomaterials for total joint replacement. J. Mech. Behav. Biomed. Mater..

[17] Perrichon A., Haochih Liu B., Chevalier J., Gremillard L., Reynard B., Farizon F., Der Liao J., Geringer J. (2017). Ageing, shocks and wear mechanism in ZTA and the long-term performance of hip joint materials. Materials.

[18] Lima M.M., Godoy C., Modenesi P.J., Avelar-Batista J.C., Davison A., Matthews A. (2004). Coating fracture toughness determined by Vickers indentation: An important parameter in cavitation erosion resistance of WC–Co thermally sprayed coatings. Surf. Coat. Technol..

[19] (2016). Standard Test Method for Linearly Reciprocating Ball-on-Flat Sliding Wear.

[20] Zhao X., An Y., Chen J., Zhou H., Yin B. (2008). Properties of Al_2_O_3_–40 wt%ZrO_2_ composite coatings from ultra-fine feedstocks by atmospheric plasma spraying. Wear.

[21] Suffner J., Sieger H., Hahn H., Dosta S., Cano I.G., Guilemany J.M., Klimczyk P., Jaworska L. (2009). Microstructure and mechanical properties of near-eutectic ZrO_2_–60 wt%Al_2_O–produced by quenched plasma spraying. Mater. Sci. Eng. A.

[22] Dejang N., Limpichaipanit A., Watcharapasorn A., Wirojanupatump S., Niranatlumpong P., Jiansirisomboon S. (2011). Fabrication and properties of plasma-sprayed Al_2_O_3_/ZrO_2_ composite coatings. J. Therm. Spray Technol..

[23] McPherson R. (1980). On the formation of thermally sprayed alumina coatings. J. Mater. Sci..

[24] Chen D., Jordan E.H., Gell M. (2009). Microstructure of suspension plasma spray and air plasma spray Al_2_O_3_–ZrO_2_ composite coatings. J. Therm. Spray Technol..

[25] Sivakumar G., Dusane R.O., Joshi S.V. (2013). A novel approach to process phase pure α-Al_2_O_3_ coatings by solution precursor plasma spraying. J. Eur. Ceram. Soc..

[26] Luo H., Goberman D., Shaw L., Gell M. (2003). Indentation fracture behavior of plasma-sprayed nanostructured Al_2_O_3_-13wt.%TiO_2_ coatings. Mater. Sci. Eng. A.

[27] Murray J.W., Ang A.S.M., Pala Z., Shaw E.C., Hussain T. (2016). Suspension High Velocity Oxy-Fuel (SHVOF)-Sprayed Alumina Coatings: Microstructure, Nanoindentation and Wear. J. Therm. Spray Technol..

[28] Hawthorne H.M., Erickson L.C., Ross D., Tai H., Troczynski T. (1997). The microstructure dependence of wear and indentation behaviour of some plasma-sprayed alumina coatings. Wear.

[29] Erickson L.C., Hawthorne H.M., Troczynski T. (2001). Correlations between micro- structural parameters, micro mechanical properties and wear resistance of plasma sprayed ceramic coatings. Wear.

[30] Normand B., Fervel V., Coddet C., Nikitine V. (2000). Tribological properties of plasma sprayed alumina–titania coatings: Role and control of the microstructure. Surf. Coat. Technol..

[31] Fervel V., Normand B., Coddet C. (1999). Tribological behaviour of plasma sprayed Al_2_O_3_-based cermet coatings. Wear.

[32] Murray J.W., Leva A., Joshi S., Hussain T. (2018). Microstructure and wear behaviour of powder and suspension hybrid Al_2_O_3_-YSZ coatings. Ceram. Int..

[33] Yan Y., Yang H., Wang L., Su Y., Qiao L. (2016). Effect of tribology processes on adsorption of albumin. Surf. Topogr. Metrol. Prop..

[34] Parkes M., Myant C., Cann P.M., Wong J.S.S. (2015). Synovial fluid lubrication: The effect of protein interactions on adsorbed and lubricating films. Biotribology.

[35] Ahmed A., Masjuki H.H., Varman M., Kalam M.A., Habibullah M., Al Mahmud K.A.H. (2016). An overview of geometrical parameters of surface texturing for piston/cylinder assembly and mechanical seals. Meccanica.

[36] Ma L., Rainforth W.M. (2012). The effect of lubrication on the friction and wear of Biolox^®^Delta. Acta Biomater..

